# The effects of cigarette smoking on the salivary and tongue microbiome

**DOI:** 10.1002/cre2.489

**Published:** 2021-09-10

**Authors:** Nao Suzuki, Yoshio Nakano, Masahiro Yoneda, Takao Hirofuji, Takashi Hanioka

**Affiliations:** ^1^ Department of Preventive and Public Health Dentistry Fukuoka Dental College Fukuoka Japan; ^2^ Oral Medicine Research Center Fukuoka Dental College Fukuoka Japan; ^3^ Department of Chemistry Nihon University School of Dentistry Tokyo Japan; ^4^ Department of General Dentistry Fukuoka Dental College Fukuoka Japan

**Keywords:** 16S rRNA gene sequencing, cigarette smoking, oral microbiome, saliva, tongue

## Abstract

**Objectives:**

It has been suggested that smoking affects the oral microbiome, but its effects on sites other than the subgingival microbiome remain unclear. This study investigated the composition of the salivary and tongue bacterial communities of smokers and nonsmokers in periodontally healthy adults.

**Methods:**

The study population included 50 healthy adults. The bacterial composition of resting saliva and the tongue coating was identified through barcoded pyrosequencing analysis of the 16S rRNA gene. The Brinkman index (BI) was used to calculate lifetime exposure to smoking. The richness and diversity of the microbiome were evaluated using the *t*‐test. Differences in the proportions of bacterial genera between smokers and nonsmokers were evaluated using the Mann–Whitney *U* test. The quantitative relationship between the proportions of genera and the BI was evaluated using Pearson's correlation analysis.

**Results:**

The richness and diversity of the oral microbiome differed significantly between saliva and the tongue but not between smokers and nonsmokers. The saliva samples from smokers were enriched with the genera *Treponema* and *Selenomonas*. The tongue samples from smokers were enriched with the genera *Dialister* and *Atopobium*. The genus *Cardiobacterium* in saliva, and the genus *Granulicatella* on the tongue, were negatively correlated with BI values. On the other hand, the genera *Treponema*, *Oribacterium*, *Dialister*, *Filifactor*, *Veillonella*, and *Selenomonas* in saliva and *Dialister*, *Bifidobacterium*, *Megasphaera*, *Mitsuokella*, and *Cryptobacterium* on the tongue were positively correlated with BI values.

**Conclusions:**

The saliva and tongue microbial profiles of smokers and nonsmokers differed in periodontally healthy adults. The genera associated with periodontitis and oral malodor accounted for high proportions in saliva and on the tongue of smokers without periodontitis and were positively correlated with lifetime exposure to smoking. The tongue might be a reservoir of pathogens associated with oral disease in smokers.

## 1 INTRODUCTION

Periodontal disease is one of the most common chronic diseases and the cause of tooth loss among adults (Frencken et al., 2017). Tobacco smoking is recognized as the most important environmental risk factor for periodontal disease. A recent systematic review has reported that tobacco smoking increases the risk of periodontal disease by 85% (Leite et al., 2018). Smokers have deeper probing depths, greater attachment loss, more bone resorption, and fewer teeth than nonsmokers (Johnson & Hill, 2004). When implants are used, being a smoker significantly affects the failure rate, the risk of postoperative infection, and marginal bone loss (Chrcanovic et al., 2015). Cigarette smoking has a variety of effects on host‐pathogen interactions in the oral cavity, such as reduction of cell‐mediated and humoral immune responses, promotion of infection with microbial pathogens, interference with antimicrobial therapies, and strengthening of antimicrobial resistance (Barbour et al., 1997; Bateson, 1993; Epstein et al., 1993; Feldman & Anderson, 2013). Furthermore, with the development of analytical technology, many studies have been conducted on the effects of cigarette smoking on the oral microbiome. The microbiome of gingival crevicular fluid has been compared between smokers and nonsmokers in healthy individuals, patients with chronic periodontal disease, and in patients with peri‐implantitis (Mason et al., 2015; Moon et al., 2015; Tsigarida et al., 2015). Those studies have reported differences in the gingival crevicular microbiome between smokers and nonsmokers. A study that investigated the changes in microbial composition associated with stopping smoking reported that the subgingival microbial community was recolonized by a greater number of health‐associated species following nonsurgical periodontal therapy and cessation of smoking (Delima et al., 2010).

Cigarette smoke affects not only the gingival sulcus but also bacteria on the tongue, buccal mucosa, and plaque. However, the effects of smoking on sites other than the gingival crevicular fluid have rarely been investigated, except in studies conducted on oral wash samples (Wu et al., 2016) and buccal mucosa (Yu et al., 2017). Investigating the effect of smoking on the microbial ecosystems in various sites of the oral cavity is important for developing an oral health strategy. The tongue occupies a large area in the oral cavity and has a different microbial community than the periodontal pockets and dental plaque (Simón‐Soro et al., 2013). The tongue coating is an important cause of oral malodor (Scully et al., 1997), and differences in tongue microbiomes with and without oral malodor have been reported (Bernardi et al., 2020). However, the effect of cigarette smoking on the tongue microbiome has not been investigated.

Therefore, this study investigated the differences in the microbial composition of the tongue directly exposed to cigarette smoke in smokers with that of nonsmokers. As the tongue microbiome is affected by periodontal disease (Tanaka et al., 2004), healthy young people without periodontal disease were targeted. In addition, the study subjects did not have periodontal pockets; therefore, resting saliva was sampled to investigate the microbiota existing around the gingival sulcus.

## 2 MATERIALS AND METHODS

### 2.1 Study population

The study population comprised 50 healthy volunteers (39 men and 11 women; mean age 25.6 ± 2.1; range 23–31 years). Dental and health checkups were conducted before collecting samples. Periodontal status was assessed using the community periodontal index probe. Participants scored 0 for both bleeding on probing and probing pocket depth based on the criteria of the WHO (World Health Organization, 2013). None of the dental or health checkups detected any problems in the participants that required treatment. No participant had taken antibiotics within the prior 3 months. The study was approved by, and conducted under the supervision of, the Ethics Committee for Clinical Research of Fukuoka Gakuen (Approval No. 249). All participants understood the purpose and content of the study and provided written informed consent to participate.

The cigarette‐smoking status of the participants was determined using a self‐completed questionnaire. None of the study subjects used electronic cigarettes or smokeless cigarettes. Smoking status was defined in the questionnaire as “smoker”, an individual who had smoked ≥100 cigarettes in total after starting smoking, and “nonsmoker”, an individual who had either never smoked or had smoked <100 cigarettes in total after starting smoking (Hanioka et al., 2007). The Brinkman index (BI), which is defined as (number of cigarettes per day) × (number of years for which a person smoked) (Brinkman & Coates Jr., 1963), was used to calculate lifetime exposure to smoking.

### 2.2 Sampling

Participants were asked to collect 3 ml of resting saliva in a disposable tube at 3:30 pm at least 2.5 h after smoking, eating, or brushing their teeth. The 1‐ml whole saliva samples were pelleted through centrifugation and stored at −30°C until use. Subsequently, tongue samples were collected using the MS Tongue Cleaner (Morita, Osaka, Japan), suspended in 10 ml phosphate‐buffered saline, pelleted by centrifugation, and stored at −30°C until use.

### 2.3 16S rRNA gene sequencing analysis

DNA was extracted as described previously (Takeshita et al., 2010). Three of the saliva samples did not have sufficient bacterial DNA; therefore, 47 saliva and 50 tongue samples were investigated in this study. The V3–V4 regions of the 16S rRNA gene were amplified and sequenced on a 454 Life Sciences Genome Sequencer FLX instrument (Roche, Basel, Basel, Switzerland) from Takara Bio Inc. (Otsu, Japan).

Sequences were excluded from the analysis if they were shorter than 240 bases, and were subsequently removed if they did not include the correct primer sequence. The remaining sequences were assigned to each subject by examining the six‐base barcode sequence. UCHIME v6.1.544 (Edgar et al., 2011) was used to remove supposed chimeric sequences, and sequences with 80% of their nucleotides with fragment quality scores below 20. The remaining sequences were assigned to operational taxonomic units using CD‐HIT with a threshold of 98% pairwise identity (Li & Godzik, 2006). Rarefaction curves calculated using QIME2 (Bolyen et al., 2019) indicated that a sufficient number of reads was obtained for 16S rRNA analyses. Each sequence was compared to 1647 sequences of the 16S rRNA gene from oral bacteria deposited in HOMD (Chen et al., 2010) (HOMD 16S rRNA RefSeq Extended Version 1.1) using the BLAST algorithm, with a similarity score of 98.5% and a minimum coverage of 97% assigned to the best BLAST hit.

### 2.4 Statistical analysis

The richness and diversity of the microbiome were assessed by the number of species and the Shannon–Weiner Index, respectively. The effects of smoking on sex, age, and the richness and diversity of the microbiome were evaluated using the *t*‐test. The Mann–Whitney *U*‐test was used to compare the proportions of bacterial genera between smokers and nonsmokers. Pearson's correlation analysis was used to assess the relationships between the proportion of bacterial genera and BI values. R software (version 4.0.3) (The R project homepage, 2021) was used for all statistical analyses. The level of significance was set at *p* < 0.05.

## 3 RESULTS

### 3.1 Study population and samples

Eighteen participants (16 men and two women; mean age, 26.8 ± 2.4 years) were smokers and 32 (23 men and nine women; mean age, 25.0 ± 1.6 years) were nonsmokers. No association between sex and smoking status was found (*p* = 0.163). Smokers were older than nonsmokers (*p* = 0.006). BI values in smokers ranged from 35 to 450, and the average value (±SD) was 162.8 ± 120.4. The BI value of one patient was >400, and that patient was considered a heavy smoker. Three saliva samples–from two smokers and one nonsmoker–and two tongue samples–from one smoker and one nonsmoker–could not be analyzed because an insufficient amount of DNA was extracted from the samples.

### 3.2 Richness and diversity of the microbiome

**Figure 1:**
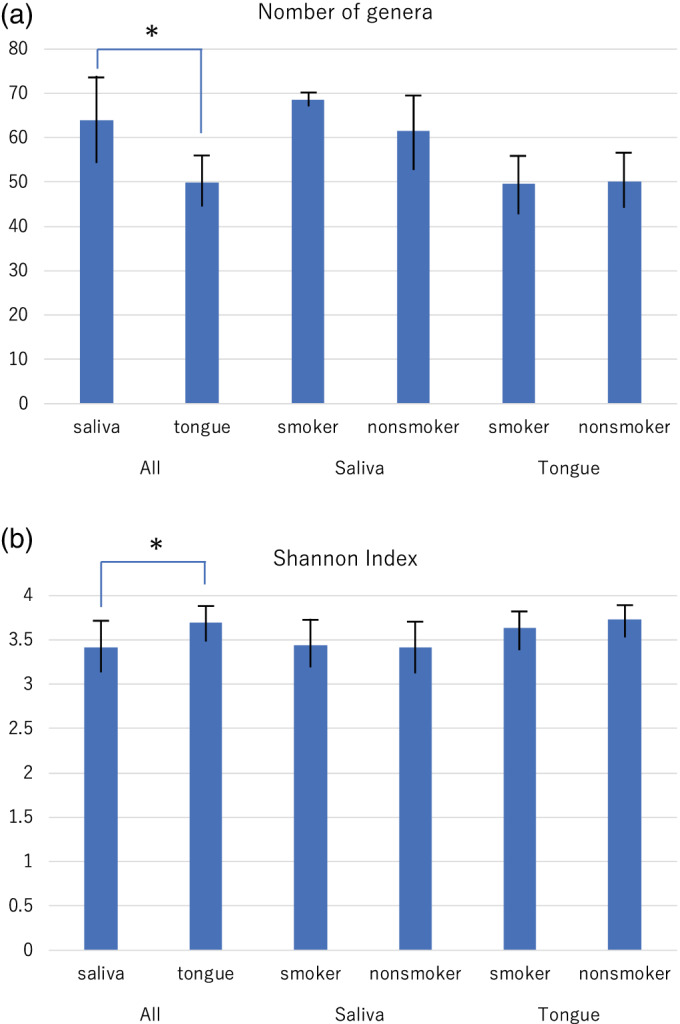
Comparison of the number of bacterial genera (a) and the Shannon index (b) Between the saliva and tongue, and between smokers and nonsmokers in the saliva, and on the tongue. *p < 0.05 between saliva and tongue according to *t*‐test

In total, 99 bacterial genera and 228 species were detected in the saliva and tongue samples. The average number (± SD) of species in the saliva was 64.0 ± 19.5, and the number on the tongue was 50.0 ± 10.7 (Figure 1a *p* = 0.000). By contrast, the average number of species in the saliva was 68.5 ± 2.12 in smokers and 61.5 ± 17.8 in nonsmokers, while the tongue hosted 49.6 ± 10.9 species in smokers and 50.2 ± 10.8 in nonsmokers (Figure 1a *p* < 0.05). Subsequently, the difference in the overall phylogenetic community between smokers and nonsmokers was assessed using the Shannon–Wiener Index. A distinct overall bacterial community composition was observed in saliva (3.42 ± 0.53) and on the tongue (3.69 ± 0.44) (Figure 1b *p* = 0.013). No significant differences in the diversity of the bacterial communities between smokers and nonsmokers were observed in saliva or on the tongue (Figure 1b).

### 3.3 Comparison of the proportions of bacterial genera in smokers and nonsmokers

**Figure 2:**
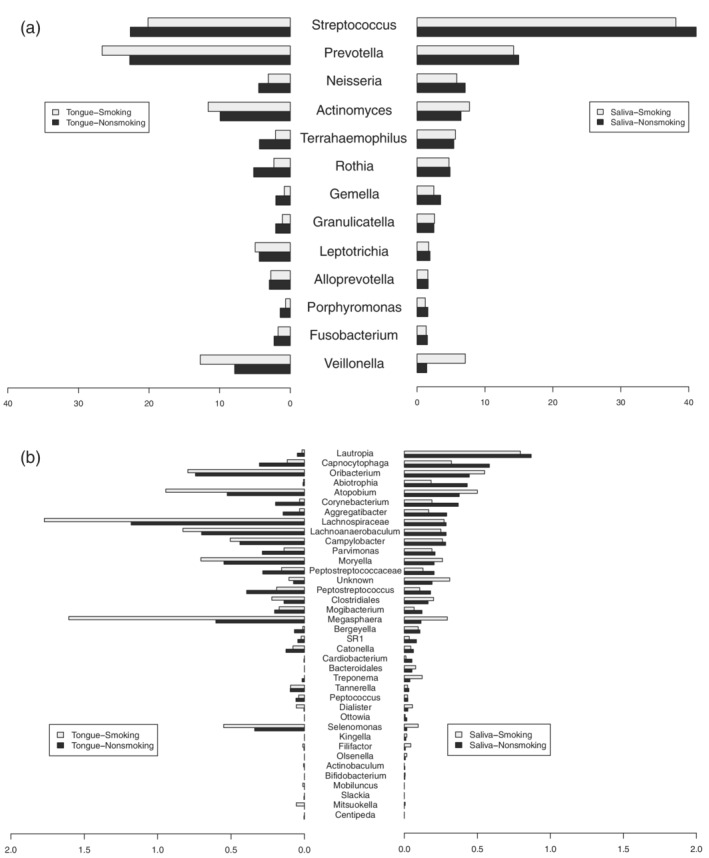
Comparison of the proportion of bacterial genera between smokers and nonsmokers. Genera with high proportions (a) and genera with low proportions (b). The left side is the tongue microbiome and the right side is the saliva microbiome. Light‐gray bars show the proportions of genera in smokers, and black bars show the proportions of genera in nonsmokers

**Figure 3:**
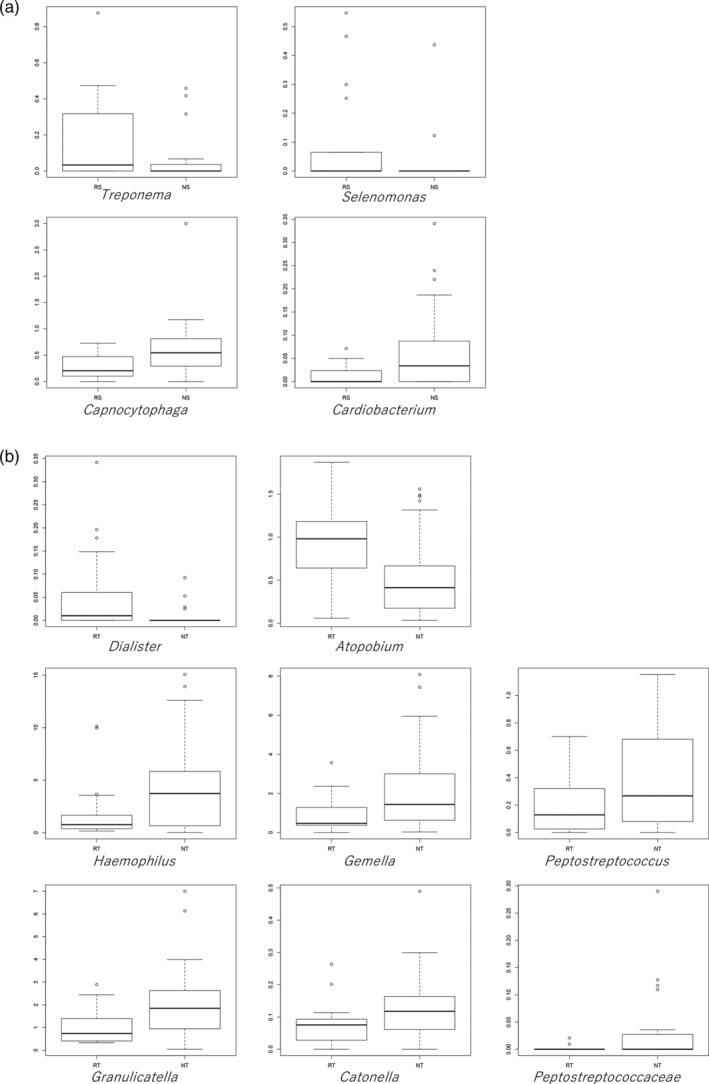
Bacterial genera in saliva (a) and on the tongue (b) that differed significantly between smokers and nonsmokers

The proportions of bacterial genera were compared between smokers and nonsmokers (Figure 2). The genera *Streptococcus*, *Prevotella*, *Neisseria*, and *Actinomyces* comprised the highest proportions in the saliva microbiota, whereas the genera *Streptococcus*, *Prevotella*, *Actinomyces*, and *Veillonella* comprised high proportions in the tongue microbiota. Compared to those from nonsmokers, the saliva samples from smokers were significantly enriched in the genera *Treponema* (*p* = 0.030) and *Selenomonas* (*p* = 0.037), and depleted in *Capnocytophaga* (*p* = 0.014) and *Cardiobacterium* (*p* = 0.010) (Figure 3a). By contrast, the tongue samples from the smokers were enriched in the genera *Dialister* (*p* = 0.003) and *Atopobium* (*p* = 0.007), and depleted in *Haemophilus* (*p* = 0.044), *Gemella* (*p* = 0.008), *Peptostreptococcus* (*p* = 0.040), *Granulicatella* (*p* = 0.022), *Catonella* (*p* = 0.049), and *Peptostreptococcaceae* (*p* = 0.027) (Figure 3b).

### 3.4 Bacterial genera related to lifetime exposure to smoking

Table 1 shows the bacterial genera correlated with BI values at α < 0.05. The genus *Bifidobacterium* was positively correlated with BI values in the tongue samples (r = 0.680). The genus *Dialister* was positively correlated with BI values in the saliva and tongue samples. The genera *Cardiobacterium* and *Granulicatella* were negatively correlated with BI values in saliva and the tongue, respectively. The genera *Treponema* and *Selenomonas*, which were predominant in the saliva of smokers compared with nonsmokers, were positively correlated with BI values in the saliva samples.

**Table 1:** Pearson's correlation coefficients between the relative abundances of the oral bacterial genera and Brinkman index values (α < 0.05)

Saliva (*n* = 47)		Tongue (*n* = 48)	
Genus	r	Genus	r
*Treponema*	0.366	*Granulicatella*	−0.282
*Cardiobacterium*	−0.292	*Dialister*	0.416
*Oribacterium*	0.324	*Bifidobacterium*	0.680
*Dialister*	0.300	*Megasphaera*	0.295
*Filifactor*	0.373	*Mitsuokella*	0.522
*Veillonella*	0.336	*Cryptobacterium*	0.437
*Selenomonas*	0.369		

*Note*: r, Pearson's correlation coefficient. Genera with three or fewer detected samples were omitted.

## 4 DISCUSSION

This is the first study to report the effects of smoking on the microbiome of resting saliva and the tongue. The bacterial diversities of the different oral micro‐niches are dependent on location (Simón‐Soro et al., 2013). The effect of smoking on the microbiome is also expected to differ from site to site. Many studies have examined the effects of smoking on the microbiome in gingival crevicular fluid (Mason et al., 2015; Moon et al., 2015; Tsigarida et al., 2015; Delima et al., 2010), and a few studies have examined the microbiome in mouth‐rinse water and buccal mucosa (Wu et al., 2016; Yu et al., 2017; Morris et al., 2013). Studies investigating microbiomes based on mouth‐rinse samples and bronchoscopic alveolar lavage samples have reported differences in the oral microbiomes of smokers, although the lung microbiomes did not differ significanlty (Morris et al., 2013). 16S rRNA sequencing of supra‐ and subgingival dental plaque, saliva, soft oral tissue, and nasal swab samples has revealed lower alpha diversity in smokers than in nonsmokers in the buccal mucosa, whereas samples from other sites did not differ significantly in microbial diversity or composition (Yu et al., 2017). These findings indicate that the oral microbiome is potentially susceptible to smoking. The current study found that microbial diversity differed significantly between resting saliva and the tongue coating, but there was no significant difference in the microbial diversity of saliva or on the tongue between smokers and nonsmokers, and some predominant genera in smokers were found at the genus level. Other studies have reported significant differences in the microbial diversity of subgingival plaque (Mason et al., 2015) and oral wash samples (Wu et al., 2016) between smokers and nonsmokers. The participants in the current study were young and had healthy periodontal tissues; therefore, no differences were observed. However, it is noteworthy that there were generic‐level microbial differences between smokers and nonsmokers, even though the subjects had no illness or symptoms. In particular, periodontopathic bacteria and the organisms relative to oral malodor increase were found in higher proportions in smokers.

The tongue is the most important anatomical structure in the oral cavity due to its location and functions (Roldán et al., 2003). Oral microorganisms existing on the tongue dorsum have easy access to nutrients, including saliva, epithelium, and food debris (Roldán et al., 2003). The tongue coating is an important source of volatile sulfur compounds, the main component of oral malodor (Scully et al., 1997). It has also been suggested to function as a reservoir for periodontopathic pathogens (Tanaka et al., 2004). Tongue morphology is reported to be negatively affected by smoking (Konstantinidis et al., 2010). Hence, it was strongly predicted that the tongue microbiota would be affected and changed by smoking. Major species on the tongue coating were *Streptococcus*, *Prevotella*, *Actinomyces*, and *Veillonella* in the present study, which is similar to previous reports that investigated the bacterial composition of the tongue dorsum (Aas et al., 2005; Washio et al., 2005). Washio et al. (Washio et al., 2005) identified differences in the numbers of hydrogen‐sulfide‐producing bacteria, including *Prevotella*, *Actinomyces*, and *Veillonella*, between subjects with and without oral malodor, while the bacterial community of the tongue had similar compositions in the two groups. Notably, the proportions of these species tended to be higher in smokers than in nonsmokers in this study, although the difference was not significant (Figure 2). Furthermore, the proportions of *Atopobium* and *Dialister* species, which have been reported as oral malodor‐related species in previous reports, were significantly higher in the tongue samples from smokers than in those from nonsmokers (Figure 3) (Kazor et al., 2003; Takeshita et al., 2012). Our previous study investigated species in the hydrogen‐sulfide‐dominant group and the methyl‐mercaptan‐dominant group in subjects with oral malodor, and the proportions of *Atopobium* and *Dialister* species were higher in the methyl‐mercaptan‐dominant group than the no‐odor group (Takeshita et al., 2012). The levels of these species increase in the subgingival plaque of patients with chronic periodontal disease (Kumar et al., 2005).

The proportions of the genera *Treponema* and *Selenomonas* in resting saliva were significantly higher in smokers than in nonsmokers (Figures 2 and 3). Furthermore, those organisms were positively correlated with BI values (Table 1). Resting saliva has been reported to have a microbial composition that differs from that of other sites in the oral cavity (Simón‐Soro et al., 2013), indicating that it represents some bacteria that do not colonize the teeth, gingival sulcus, or tongue. The current study detected a significant difference in microbial diversity between resting saliva and the tongue (Figure 1). The genera *Treponema* and *Selenomonas* are motile bacilli related to aggressive periodontitis and oral malodor. Both are potent hydrogen sulfide producers in the presence of L‐cysteine (Persson et al., 1990). However, *Selenomonas* species were significantly more predominant in the methyl‐mercaptan‐dominant group than the no‐odor group in our previous study (Takeshita et al., 2012). The increases in these organisms in resting saliva imply inflammation of the gingival crevice.

Most genera that were positively correlated with BI values were strictly anaerobic and have been reported to be periodontitis‐ and oral‐malodor‐associated microorganisms (Table 1). The quantitative relationship between these genera and tobacco exposure is supported by previous studies, in which 12 months of smoking cessation reduced the proportions of *Treponema* and *Dialister* in subgingival plaques (Delima et al., 2010). *Bifidobacterium* was positively correlated with the amount of smoke on the tongue. A recent investigation using mouth‐rinse samples reported that the genus *Bifidobacterium* is enriched among current‐smokers compared with never‐smokers (Yang et al., 2019). The genera *Bifidobacterium*, *Megasphaera*, and *Mitsuokella* are adapted to low‐pH conditions (Russell, 1991; Levine et al., 2012). It is unknown why smokers have an increased number of bacteria adapted to low‐pH conditions, but it may indirectly explain the involvement of smoking and secondhand smoke in dental caries (Hanioka et al., 2011; Jiang et al., 2019).

This was a cross‐sectional study; thus, the relationship between differences in the microbiome and future onset of periodontal disease cannot be clarified. In addition, if oral malodor could be evaluated, the current relationship between differences in the microbiome and oral malodor could have been clarified; however, oral malodor was not evaluated in this study. It would be necessary to ask the subjects to quit smoking for 12 h or more to accurately determine oral malodor because otherwise it would be affected by the smell of cigarettes.

In conclusion, our findings indicate that the microbial profiles of smokers and nonsmokers in the saliva and on the tongue differed at the generic level in healthy Japanese adults. Because of the characteristics of the genera that were common to smokers and that correlated with smoking exposure, smokers may be at risk for oral malodor and future periodontitis, even if they have a clinically normal oral cavity.

## CONFLICT OF INTEREST

The author declares there is no potential conflict of interest.

## AUTHOR CONTRIBUTIONS

Nao Suzuki designed the study, collected and analyzed the data, and wrote the manuscript. Yoshio Nakano analyzed and interpreted the data, and wrote the Materials and methods and Results sections of the manuscript. Masahiro Yoneda and Takao Hirofuji were mainly involved in writing the Discussion section. Takashi Hanioka mainly wrote the Introduction and Discussion. All authors approved the final manuscript and take responsibility for its contents.

## Data Availability

The data that support the findings of this study are available from the corresponding author, N. S., upon reasonable request.
